# Dysregulation of follicle development in a mouse model of premature ovarian insufficiency

**DOI:** 10.1530/REP-16-0091

**Published:** 2016-10-11

**Authors:** P Grasa, S Sheikh, N Krzys, K Millar, S Janjua, P Nawaggi, S A Williams

**Affiliations:** 1 Nuffield Department of Obstetrics and GynaecologyUniversity of Oxford, Women’s Centre, Level 3, John Radcliffe Hospital, Oxford, UK; 2 Department of PhysiologyAnatomy and Genetics, University of Oxford, Oxford, UK

## Abstract

Premature ovarian insufficiency (POI) occurs in 1% of reproductive-age women. The ovarian manifestation ranges from the presence of a variable population of follicles (follicular) to the absence of follicles (afollicular), and in the majority of cases the cause is unknown. A transgenic mouse model of follicular POI, the Double Mutant (DM), arises from oocyte-specific deletion of *Mgat1* and *C1galt1* required for the generation of *O*- and *N*-glycans. DM females are subfertile at 6 weeks, infertile by 9 weeks and exhibit POI by 12 weeks of age. In this study we investigate the cause of the reduced fertility at 6 weeks and infertility at 9 weeks of DM females. Ovary sections were used to analyse follicle and corpora lutea (CL) numbers, apoptosis, and levels of laminin and 3β-hydroxysteroid dehydrogenase using immunohistochemistry. After POI, DM females unexpectedly remained sexually receptive. At both 6 and 9 weeks, DM ovaries contained more primary follicles, however, at 9 weeks DM follicles were proportionally healthier, revealed by TUNEL analysis compared with Controls. In 9 week DM ovaries (collected post-mating), secondary follicles had theca and basal lamina structure abnormalities, whilst preovulatory follicles failed to ovulate resulting in the presence of numerous luteinised unruptured follicles, indicative of ovulation failure. Finally, DM ovaries contained more regressing CL with decreased luteal cell apoptosis indicative of a defect in CL regression. Identifying these follicular modifications have provided insight into the aetiology of a model of POI and highlight targets to investigate with the hope of developing new fertility treatments.

## Introduction

Follicle development starts with the activation and development of quiescent primordial follicles that develop through primary, secondary, preantral and antral follicle stages before they ovulate a fertilisable oocyte, however, many follicles undergo atresia and die (Greenwald 1972, Hirshfield 1991). At the primary follicle stage, the oocyte and its supporting cuboidal granulosa cells (GCs) are encapsulated by a basal lamina (BL) composed of extracellular matrix (ECM). The BL maintains the integrity of the follicle and influences follicle growth by regulating the molecules that enter the follicle (Rodgers *et al.* 1999, Irving-Rodgers *et al.* 2010). At the secondary follicle stage, theca cells are recruited from the surrounding stromal tissue (Young & McNeilly 2010) and the GCs proliferate, resulting in the formation of a preantral follicle. This preantral follicle develops an antrum to become an antral follicle, which develops further to become a large preovulatory follicle from which the mature oocyte will ovulate. Complex interactions between the oocyte, the granulosa and theca cells orchestrate follicle development with oocyte-specific proteins and glycoproteins playing an active role through their interaction with the surrounding granulosa and cumulus cells (Eppig 2001, Matzuk *et al.* 2002, Williams & Stanley 2011, Christensen *et al.* 2015, Grasa *et al.* 2015, Ploutarchou *et al.* 2015). After ovulation, the follicular cells differentiate to form a highly vascularised endocrine organ, the corpus luteum (CL), whose primary function is to produce progesterone to support the ensuing pregnancy. If, however, pregnancy does not occur, the CL regresses (Nelson *et al.* 1982, Carambula *et al.* 2002, Davis & Rueda 2002).

Abnormal control of primordial follicle activation or development can lead to conditions such as premature ovarian insufficiency (POI), previously known as premature ovarian failure, which affects 1–3% of women under 40 years of age and is idiopathic in over 70% of cases (Coulam *et al.* 1986, Meskhi & Seif 2006, Shelling 2010). There are two main aetiological mechanisms, which give rise to a heterogeneous spectrum of ovarian manifestations. The first is follicle depletion resulting in ovaries devoid of follicles (afollicular POI) and the second mechanism is follicle dysfunction, resulting in a spectrum of follicle development, from the presence of a variable population of follicles, including antral follicles, that fail to develop or the presence of only primordial follicles (follicular POI) (Nelson *et al.* 1994, Meskhi & Seif 2006, Nelson 2009, Hubayter *et al.* 2010, Suzuki *et al.* 2015). Follicular POI accounts for around 50% of cases (Mehta *et al.* 1992, Nelson *et al.* 1994, Massin *et al.* 2004, Suzuki *et al.* 2015). Although several factors have been related with POI, in the majority of cases the causal mechanism remains unclear, and further research using POI models is therefore warranted.

A mouse model of follicular POI has been established (Williams & Stanley 2011); known as the Double Mutant (DM). The genetic origin of the DM mouse is oocyte-specific deletion of two glycosyltransferase genes, *C1galt1* (also known as *T-syn*) and *Mgat1*, that respectively encode β1,3-galactosyltransferase (T-synthase) and *N*-acetylglucosaminyltransferase I (GlcNAcT-1). T-synthase is required for the generation of core 1-derived *O*-glycans (Ju *et al.* 2002*a*,*b*), whilst GlcNAcT-1 is required for the synthesis of complex and hybrid *N*-glycans (Schlesinger *et al.* 1975, Robertson *et al.* 1978). Oocyte generated *O-* and *N-*glycans are involved in the regulation of multiple aspects of follicle development and ovarian function such as the formation of the BL and theca cells, and cumulus expansion (Williams & Stanley 2008, 2009, Grasa *et al.* 2012, 2015, Christensen *et al.* 2015, Ploutarchou *et al.* 2015).

DM female mice are subfertile at 6 weeks, infertile at 9 weeks and undergo POI by 3 months of age (Williams & Stanley 2011, Grasa *et al.* 2012). This drop in fertility of DM females is accompanied by a dysregulation of follicle development and an altered endocrine profile at 3 months (Williams & Stanley 2011). Although DM females have decreased fertility at 6 weeks, they have a normal ovulation rate, however, DM ovaries contain a larger and more heterogeneous population of CL (Grasa *et al.* 2012). By 3 months of age, DM ovaries lack developing follicles and abnormal luteinised structures are present (Williams & Stanley 2011). Therefore, the clearly defined onset of POI in the DM makes this an excellent model to study the potential aetiology of POI. In this study, we investigate the onset and aetiology of POI in postpubertal DM females, by studying the reproductive phenotype and ovarian function.

## Materials and methods

### Mice

DM mice are homozygous for floxed *Mgat1* and *C1galt1* alleles and carry a *ZP3Cre recombinase* transgene (Williams *et al.* 2007). The floxed alleles are deleted when exposed to *Cre* recombinase, expression of which is controlled by the promoter of oocyte-specific *ZP3*, which is expressed only in the developing oocyte from the primary stage of follicle development (Philpott *et al.* 1987). Experimental females (*Mgat1*^F/F^*C1galt1*^F/F^:*ZP3Cre*) carry floxed alleles of *Mgat1* and *C1galt1* and a *ZP3Cre* recombinase transgene, whilst Control females lack the *ZP3Cre* transgene (*Mgat1*^F/F^*C1galt1*^F/F^) (Williams *et al.* 2007, Williams & Stanley 2011). The *ZP3Cre* transgene does not affect fertility (Shi *et al.* 2004, Williams *et al.* 2007).

Mice were maintained in individually ventilated cages in 12:12 h light–darkness cycles unless specified otherwise.

### Ethical approval

All experiments using mice were carried out with the approval of the Local Ethical Review Panel at the University of Oxford under licence in accordance with the UK Animals (Scientific Procedures) Act 1986.

### Genotyping

Mice were genotyped using protocols as described previously (Grasa *et al.* 2012, 2015).

### Reproductive parameters and oestrous cycle evaluation

To assess sexual receptivity, Control and DM females at 6 weeks of age were caged with males and mating assessed daily (confirmed by the presence of a vaginal plug), until 6 months of age. To evaluate the oestrous cycle, vaginal smears were obtained daily between 08:00 and 10:00 from Control and DM females that were caged together in open top cages in close proximity to males from 4 weeks to 6 months of age. Cell smears were stained with Giemsa (Sigma-Aldrich) and assessed to determine the four stages of the oestrous cycle as described previously (Grasa *et al.* 2015).

### Ovarian histology and follicle counts

To ensure ovaries were collected at the same stage of the oestrous cycle, ovaries were collected on the day after mating from females put together with males at 6 or 9 weeks of age. Ovaries were collected from females that mated within 7 days of joining. Ovaries were weighed, fixed in 10% buffered formalin (Sigma-Aldrich) for 8 h, paraffin embedded, 5 µm sections collected and mounted on glass slides.

To determine follicle numbers, every 10th serial section was stained with haematoxylin (Shandon Gill 2 Hematoxylin; Thermo Fisher Scientific) and eosin (Sigma-Aldrich) (H&E) for analysis. Sections were visualised using a DM2500 Leica microscope (Microscope Services Ltd, Woodstock, UK) and imaged using a MicroPublisher 5.0 RTV camera (Qimaging, Microscope Services Ltd). Only morphologically healthy follicles with a central oocyte and a visible nucleus were assessed. Follicles were classified as described by Grasa *et al.* (2015). For analysis, follicles were grouped into four categories: primary (3a and 3b), secondary (type 4), preantral (5a and 5b) and antral (including 5a + A, 5b + A (which develop an early antrum (A)), 6 and 7). The number of luteinised unruptured follicles (LUFs) and CL were also recorded. LUFs are large antral follicles that contain an oocyte and show signs of GC luteinisation, these were only present in post-mating ovaries at 9 weeks. To identify and record the number of CL present in the ovaries, CL were followed through serial sections (every 50 µm) of the entire ovary, thus avoiding miscounting.

### Immunohistochemistry

Immunohistochemistry (IHC) was performed on 5 µm paraffin-embedded sections to detect laminin and 3β-hydroxysteroid dehydrogenase (3BHSD). Sections were dewaxed with xylene and rehydrated using decreasing concentrations of ethanol in ddH_2_O. Slides were washed in tris buffered saline (TBS: 0.1 M Tris pH 7.5 and 0.3 M NaCl) with 0.05% Tween 20 (TBST). Endogenous peroxidase was quenched using 3% H_2_O_2_ (Thermo Fisher Scientific) in PBS for 5 min. Non-specific primary antibody binding was blocked using goat serum in TBS (NGS: Vectastain ABC Elite Kit, Vector Laboratories, Peterborough, UK). Primary antibodies were diluted in the blocking solution and incubated overnight at 4°C. The rabbit anti-laminin (L9393; Sigma) was used at 1:500 and anti-3BHSD (generously donated by Prof. Ian Mason from the University of Edinburgh) was used at 1:2000 dilution; blocking solution was used as a negative control. After three washes with TBST, sections were incubated with biotinylated anti-rabbit IgG secondary antibody (Vectastain ABC Elite Kit) for 1 h, followed by ABC solution (Vectastain ABC Elite Kit) for 30 min. A 3,3′-diaminobenzidine peroxidase substrate kit (Vector Labs) was used to visualise localisation. The slides were counterstained with haematoxylin, dehydrated, mounted with DEPEX (VWR, Leicestershire, UK) and imaged. Follicles were classified as positive or negative for the presence of 3BHSD in GCs, whilst laminin detection was classified as low or high. The number of theca cell layers was counted and the depth of theca layer measured using ImageJ (National Institutes of Health, Bethesda, Maryland, USA).

### CL regression evaluation

To assess CL regression, ovaries were collected from Control and DM females at 6 weeks of age, fixed, sectioned and stained with H&E. The number of CL present was recorded and the area of each CL was determined in the largest cross section using ImageJ software. CL were classified as Newly Formed, Type I regressing or Type II regressing based on the morphology and cellular type present. Newly formed CL were composed of luteal cells with a small amount of basophilic cytoplasm, and may contain a central fluid-filled cavity. For analysis, regressing CL were classified as Types I and II; Type I regressing CL contained luteal cells with abundant eosinophilic cytoplasm, distinct cell borders and indistinct interstitial cells whilst Type II regressing CL were smaller with a more conspicuous interstitium.

### TUNEL assay

Apoptosis of CL was assessed in formalin fixed ovarian sections from 6 week Control and DM females using the TUNEL assay (ApopTag kit; Merck Millipore, Watford, Hertfordshire, UK) as described by Grasa *et al.* (2015). Only follicles sectioned through the oocyte were analysed and assessed as being either healthy, containing low or high levels of apoptosis, or dead. Healthy follicles had no apoptotic cells and a healthy oocyte. Follicles classified with low levels of apoptosis had some apoptotic cells and a healthy oocyte. Follicles classified with high levels of apoptosis had numerous apoptotic cells but an intact follicle structure. Dead or dying follicles had lost their follicular and/or oocyte structure, large amounts of apoptosis in GCs and oocyte blebbing. To quantify the level of apoptosis in CL, mean pixel intensity of TUNEL staining of luteal cells of Type I and II regressing CL was determined using ImageJ.

### Statistical analyses

Statistical analysis was carried out using GraphPad Prism software (GraphPad Software, version 4.0b, 2004). D’Agostino–Pearson normality test or Shapiro–Wilk normality test was applied to test Gaussian (normal) distribution of the samples. The Mann–Whitney *U* test was performed to detect differences between groups with non-parametrical distribution and equal variances. Unpaired *T* test was used to analyse samples with normal distribution. A one-way ANOVA followed by multiple comparison tests were used to analyse the mean number of follicles per ovary. Results are presented as mean ± s.d./s.e.m. *P* ≤ 0.05 was considered significant.

## Results

### DM females show normal oestrous cycles and sexual receptivity

Since DM females have a normal ovulation rate but decreased fertility at 6 weeks of age, which declines dramatically by 9 weeks of age, and are infertile by 3 months of age (Williams *et al.* 2007, Williams & Stanley 2011, Grasa *et al.* 2012), we investigated the aetiology of POI. The time to first mating (Control 4.8 ± 1.1 days *n* = 4, DM 2.3 ± 0.8 days *n* = 4) or first litter (Control 27.6 ± 2.5 days *n* = 5, DM 24.0 ± 1.0 days *n* = 4) did not differ between Control and DM females consistent with our previous studies of this model (Williams & Stanley 2011, Grasa *et al.* 2012). The age at first oestrous (i.e. puberty), as detected by the presence of cornified cells, also did not differ between Control (35.0 ± 1.3 days, *n* = 9) and DM (35.3 ± 1.6 days, *n* = 8) females.

**Figure 1:**
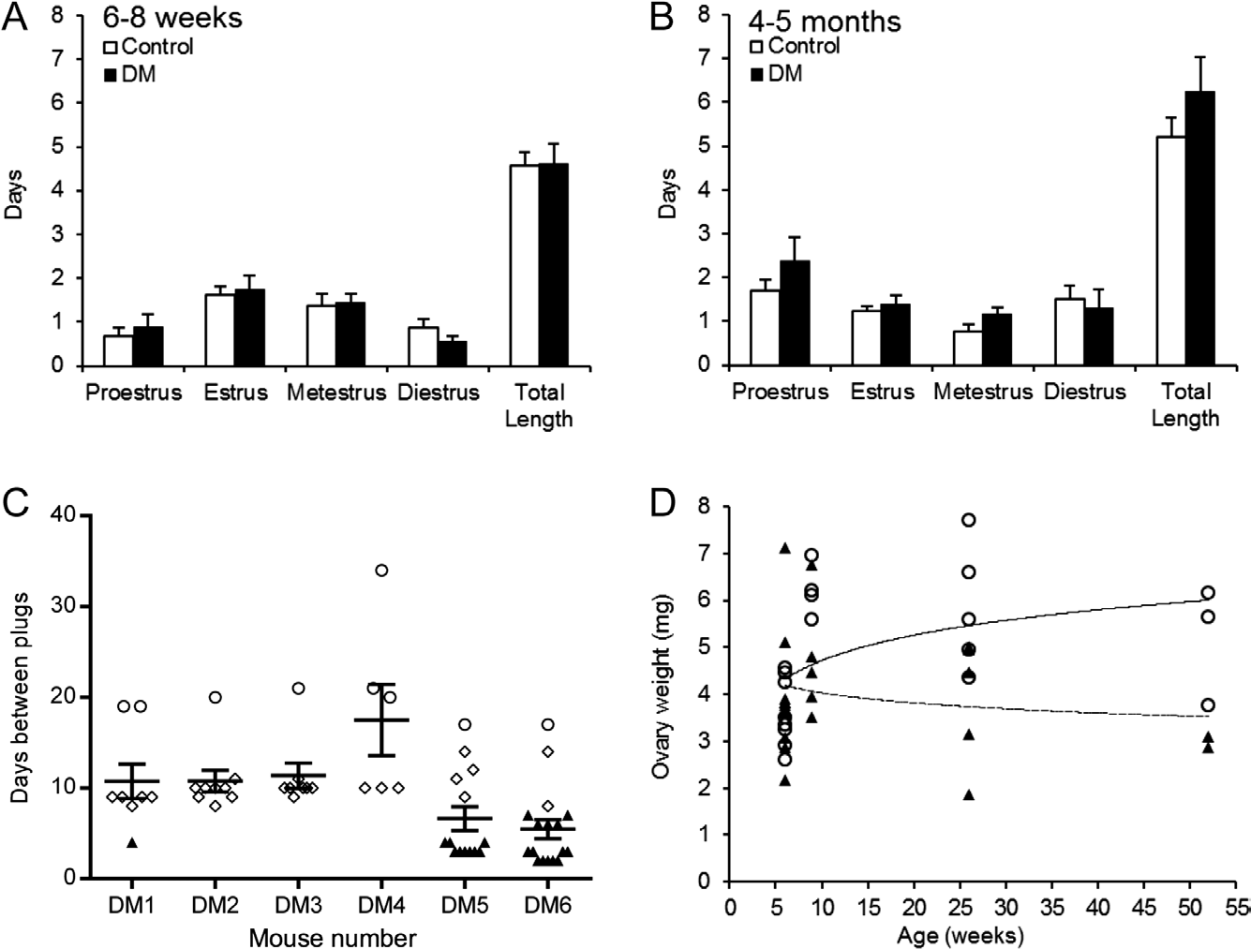
Reproductive parameters. (A and B) Oestrous cycle length. The oestrous cycle was evaluated in Control and DM females at (A) 6–8 weeks and (B) 4–5 months of age by cytological analysis of vaginal washes. (C) Interval between mating plugs of DM females. DM females were caged with males and mating plugs were checked daily from 4 weeks to 6 months of age. Control littermates had a litter every 3 weeks and therefore, are not shown here. All DM females mated repeatedly throughout the study period. Half of the DM females had plug intervals of ~4 days (black triangle; consistent with the duration of the oestrous cycle), all DM females had interplug periods of 8–14 days indicative of pseudopregnancy (open diamonds), and all exhibited interplug intervals of 17–34 days (open circles) where they appeared pregnant but did not litter down (resorption of small litters occurs in mice). (A–C) Results are expressed as mean ± s.e.m. (D) Average ovary weight of Control (open circles) and DM (black triangles) females from 6 weeks to 1 year of age. Trend line of Control (solid line) and DM (broken line) ovary weight.

Oestrous cycle parameters were evaluated between 6 and 8 weeks of age, when DM females are fertile and ovulate, and 4–5 months of age, after POI has occurred and they are infertile. There were no differences in oestrous cycle length between Control and DM females at either of the time points (Fig. 1A and B). Although DM females did not have any litters after the first litter at 9 weeks of age they remained sexually receptive as all DM females mated frequently throughout the study period; however, the period between the plugs could be divided into three separate groups (Fig. 1C). The first interplug period was ~4 days consistent with a normal oestrous cycle (Fig. 1C, black triangles), the second was an interplug interval of 8–14 days which is consistent with pseudopregnancy (Fig. 1C, open diamonds) and the third, was a longer interplug interval of 17–34 days (Fig. 1C, open circles) where females often appeared pregnant but never gave birth; we hypothesise that these females became pregnant with small litters which were resorbed.

The weight of DM and Control ovaries from mice from 6 weeks to 1 year of age were compared. An age-dependent decrease in DM ovary weight was observed whereas ovary weight increased with age in Controls (Fig. 1D).

### Follicular development in DM postpubertal females is altered

**Figure 2:**
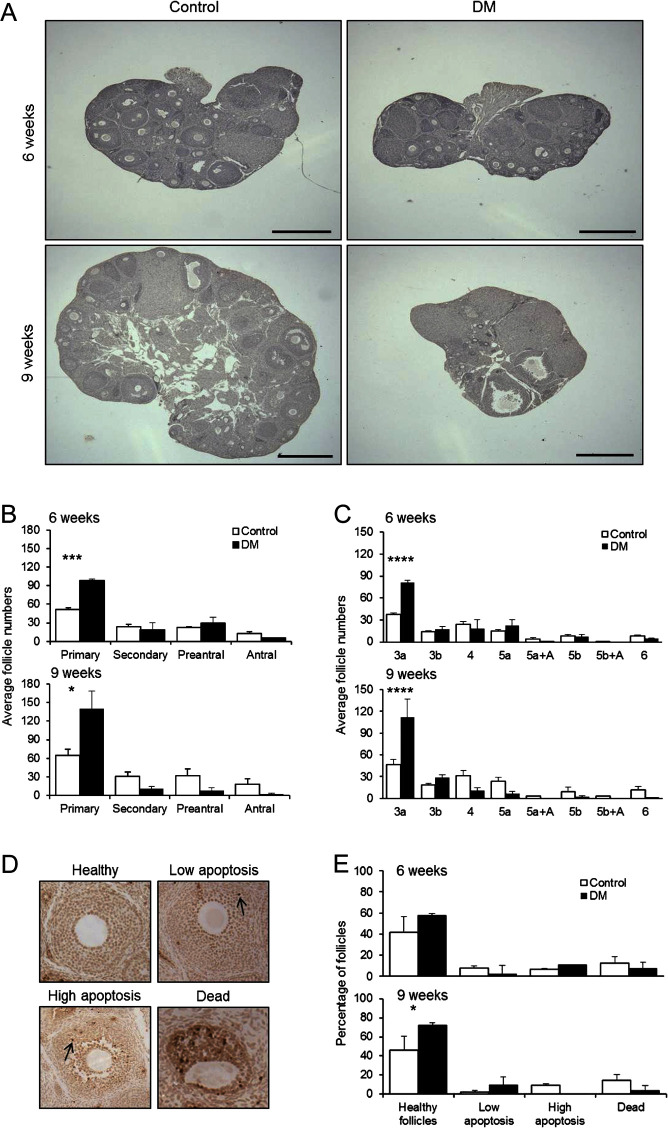
Assessment of follicle development in 6- and 9-week-old mouse ovaries. (A) Morphology of ovaries collected the day after ovulation at 6 and 9 weeks. Scale bar 500 μm. (B) Average number of follicles at the primary, secondary, preantral and antral stages of development from 6- to 9-week-old Control (*n* = 3 mice) and DM (*n* = 3 mice) ovaries. (C) Average number of follicles at each discrete stage of development (3a, 3b, 4, 5a, 5a + A, 5b, 5b + A and 6) as per Grasa *et al.* (2015) from 6 and 9-week-old Control (*n* = 3 mice) and DM (*n* = 3 mice) ovaries collected the day after ovulation. (B–D) Results are expressed as mean ± s.e.m. (D) Representative images of TUNEL staining scale used to assess levels of apoptosis in follicles. Arrow shows apoptotic cells. (E) Levels of apoptosis detected in follicles at 6 weeks of age and (Controls *n* = 96 follicles; *n* = 3 mice, DM *n* = 90 follicles; *n* = 3 mice) 9 weeks of age (Controls *n* = 116 follicles; *n* = 3 mice, DM *n* = 34 follicles; *n* = 3 mice). Results are expressed as mean ± s.d. *P ≤ 0.05, ***P ≤ 0.001, ****P ≤ 0.0001.

As reported previously, there is a dramatic decrease in the number of developing follicles in ovaries of 3-month-old DM females compared with 3-week DM or Control females (Williams & Stanley 2011). To investigate the decline in follicle numbers and thus the onset of ovarian failure, follicle development was evaluated (in ovaries collected after mating to ensure cycle synchronisation) at two time points, when females were subfertile at ~6 weeks (Control 6.5 ± 0.1 weeks; *n* = 3, DM 6.4 ± 0.1 weeks; *n* = 3) and infertile at ~9 weeks (Control 9.4 ± 0.0 weeks; *n* = 3, DM 9.4 ± 0.1 weeks; *n* = 3) (Fig. 2) (hereafter referred to as 6 weeks and 9 weeks). Control and DM ovaries at both 6 and 9 weeks contained many follicles whilst DM ovaries at 9 weeks also contained abnormal large antral follicles with oocytes missing a ZP and a thickened granulosa layer (Fig. 2A). At both 6 and 9 weeks of age, there were significantly more primary follicles in the DM ovaries when compared with Controls (Fig. 2B). Further breakdown of follicle stages into subgroups revealed that DM ovaries contained more 3a follicles with an increase of 116% at 6 weeks and 140% at 9 weeks (Fig. 2C), which is consistent with the previously reported findings at 3 months (Williams & Stanley 2011).

### DM ovaries at 9 weeks contain a higher proportion of healthier follicles

Apoptosis in follicles from 6 and 9 week ovaries was evaluated by TUNEL analysis. Follicles were classified as healthy, with low or high levels of apoptosis, or dying/dead (Fig. 2D). At 6 weeks of age there was no difference in the proportion of follicles in each of these categories between Controls and DM, however, at 9 weeks of age, of the few follicles that were present in DM ovaries, a higher proportion of these follicles were healthier compared with Controls (*P* ≤ 0.05) (Fig. 2E).

### DM follicles have a modified BL, a decreased number and depth of theca cell layers, accompanied by increased laminin content

**Figure 3:**
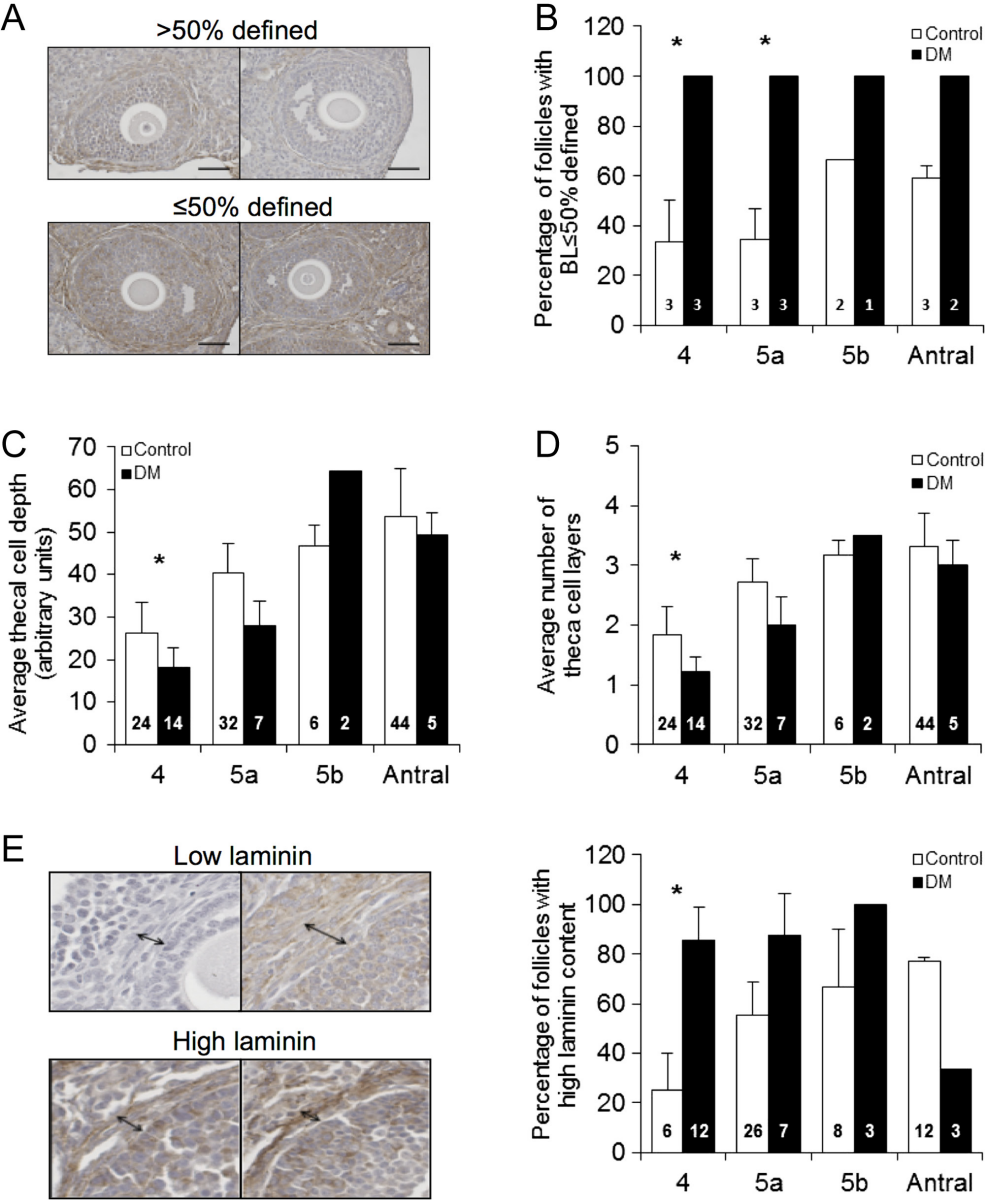
Analysis of basal lamina, theca cells and laminin by immunohistochemistry. (A) Representative images defining how follicle BL was classified as either ≤50% or >50 BL defined. (B) Proportion of Control and DM follicles at 9 weeks of age (Control *n* = 11 follicles; *n* = 3 mice, DM *n* = 9 follicles; *n* = 3 mice) classified as having less than or equal to 50% or more than 50% BL defined. (C and D) The theca cell depth and the number of theca cell layers are both reduced in DM follicles compared with Controls at 9 weeks of age (Control *n* = 106 follicles; *n* = 3 mice, DM *n* = 28 follicles; *n* = 3 mice). (E) Laminin detection in ovary sections using IHC revealed that DM follicles at the secondary stage (stage 4) have a higher laminin content in the theca compartment than Controls at 9 weeks of age. Results are expressed as mean ± s.e.m. Numbers in columns represent number of follicles. *P ≤ 0.05.

Next, we analysed the structure of the surviving follicles at 9 weeks of age, to determine if these were morphologically normal. First, integrity of the BL was assessed and follicles were classified as ≤50% defined, where the BL was too thin to identify or was indistinguishable from the surrounding stroma, or >50 defined, where the BL was clearly seen surrounding the majority of the follicle (Fig. 3A). DM had a significantly higher proportion of follicles with ≤50% defined BL at stages 4 and 5a (Control *n* = 11 follicles; *n* = 3 mice, DM *n* = 9 follicles; *n* = 3 mice) (Fig. 3B).

As reported previously, DM females have lower levels of testosterone at 3 months (Williams & Stanley 2011); therefore, we analysed the androgen generating theca cell layer at 9 weeks of age. Analyses revealed that DM follicles have a decreased theca interna depth early in development, when compared with Control follicles (Fig. 3C), which is attributed to a reduced number of theca cell layers compared with Control follicles (Control *n* = 106 follicles; *n* = 3 mice, DM *n* = 28 follicles; *n* = 3 mice) (Fig. 3D).

Finally, ECM laminin content within the thecal compartment was analysed by IHC and categorised as ‘high’, where intense staining of laminin was detected around the entire BL, or ‘low’ content, where there was faint or no visible staining. The percentage of DM follicles with high laminin content was higher at the secondary stage when compared with Controls and levels did not drop until the antral stage, whereas Control follicles exhibited a gradual increase in laminin content during development (Fig. 3E).

### The decrease in ovulation rate in DM ovaries at 9 weeks post-mating is due to premature luteinisation of preovulatory follicles

**Figure 4:**
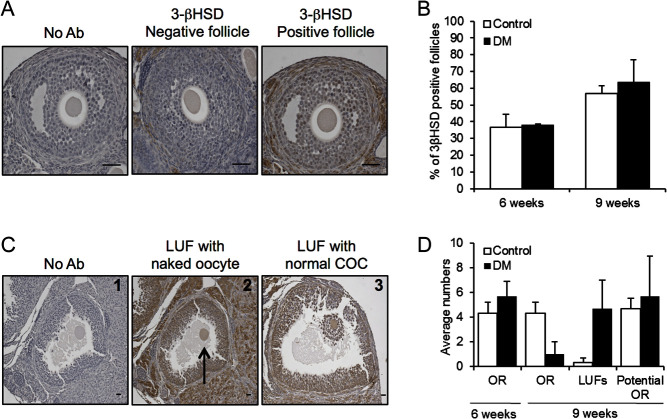
3BHSD detection in Control and DM ovarian follicles using IHC. (A) Follicles not incubated with antibody (No Ab), follicles incubated with 3BHSD antibody and were either negative for 3BHSD (3BHSD negative follicle) or 3BHSD was detected (3BHSD positive follicle) at 6 and 9 weeks of age (scale bars = 50 μm). (B) Proportion of follicles in Control and DM ovaries either positive or negative for 3BHSD detection. (C1–3) LUFs in Control and DM ovaries at 9 weeks of age. Scale bars correspond to 100 μm. (C1) Follicles not incubated with antibody. (C2) Arrow shows an oocyte free of cumulus cells in a LUF. (C3) A cumulus oocyte complex within a LUF. (D) To assess if LUFs accounted for the previously reported drop in ovulation rate (OR) in DM between 6 and 9 weeks of age, the right ovary OR for Control and DM ovaries at 6 and 9 weeks of age was assessed by number of eggs collected from the right ovary. The OR at 9 weeks was added to the number of LUFs present in the right ovary at 9 weeks to calculate the ‘potential OR’ (Control *n* = 3, DM *n* = 3). Results are expressed as mean ± s.e.m.

To explore the molecular mechanisms underlying the altered follicle development, changes in the levels of 3BHSD, a marker of steroidogenic activity, were assessed in ovaries collected after mating using IHC. Developing follicles were assessed and classified as either positive or negative for detection of 3BHSD in the GC compartment (Fig. 4A). Large unruptured preovulatory follicles containing an oocyte with GCs staining strongly positive for 3BHSD were classified as LUFs. No differences were found in the proportions of developing follicles staining positively between DM and Control ovaries at 6 weeks (Control *n* = 23 follicles; *n* = 3, DM *n* = 24 follicles; *n* = 3) or 9 weeks (Control *n* = 70 follicles; *n* = 3, DM *n* = 15 follicles; *n* = 3) (Fig. 4B). However, LUFs were detected at 9 weeks of age in both Control and DM ovaries (Fig. 4C). The oocytes of some DM LUFs lacked cumulus cells (Fig. 4C2). The number of LUFs in Control ovaries was very low, with less than one found on average; however, in DM ovaries, >4 on average were present (Fig. 4D).

To ascertain if DM females have a decreased ovulation rate, and thus fertility, at 9 weeks of age due to the premature luteinisation of preovulatory follicles, we examined the ovulation rate of the right ovary (determined by the number of eggs collected from the right oviduct) and the numbers of LUFs present in the right ovary (counted by histological analysis). Consistent with previous findings (Grasa *et al.* 2012), the ovulation rate at 6 weeks did not differ between DM (5.67 ± 2.08; *n* = 3) and Controls (4.33 ± 1.53; *n* = 3) (Fig. 4D); overall average ovulation rate per mouse did not differ between Controls and DM (both ovaries: DM 8.00 ± 3.00; *n* = 3 Control 8.33 ± 0.58; *n* = 3). Whereas at 9 weeks of age, although the right ovary ovulation rate for Controls at 9 weeks of age (4.33 ± 1.53) was consistent with right ovary ovulation rate for Controls at 6 weeks of age (4.33 ± 1.53), the DM right ovary ovulation rate had declined dramatically (1.00 ± 1.73) (*P* = 0.07) (both ovaries: DM 1.0 ± 1.73; *n* = 3 Control 9.00 ± 2.65; *n* = 3). For both Control and DM females, the ovulation rate at 9 weeks of age (assessed by number of eggs collected from the right oviduct) and the number of LUFs present in the right ovary at 9 weeks of age were combined to calculate the ‘potential ovulation rate’ (Fig. 4C). The ‘potential ovulation rate’ calculated for DM at 9 weeks of age matched that of Control and DM females at 6 weeks of age suggesting the decrease in ovulation rate at 9 weeks of age may be due to premature luteinisation of preovulatory follicles.

### DM ovaries contain more but smaller regressing CL than Control ovaries

**Figure 5:**
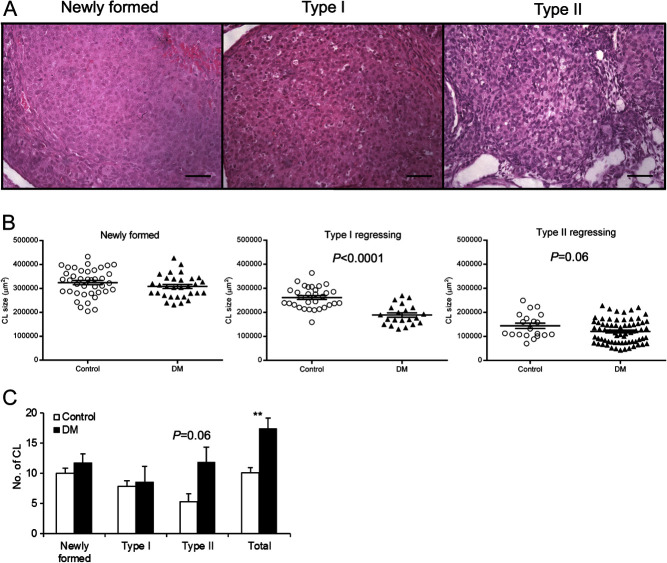
Assessment of CL regression in Control and DM ovaries at 6 weeks of age. (A) Representative images of Newly formed, Type I and II regressing CL. Scale bars correspond to 50 μm. (B) CL size of Newly formed, Type I and II regressing CL in Control (open circles) and DM (black triangles) ovaries measured in the central CL cross section. DM have smaller Type I and II regressing CL compared with Controls (Newly Formed: Control *n* = 40 and DM *n* = 31, Type I regressing: Control *n* = 31 and DM *n* = 21, Type II regressing: Control *n* = 20 and DM *n* = 69). Individual points represent data for each CL and the error bar is mean ± s.e.m. (C) Number of each type of CL present in Control and DM ovaries. NB Each female did not contain all three types of CL. Newly formed (Control *n* = 4, DM *n* = 3), Type I (Control *n* = 4, DM *n* = 3) and II (Control *n* = 3, DM *n* = 6) regressing and total CL (Control *n* = 9, DM *n* = 8). Results are expressed as mean ± s.e.m. **P ≤ 0.01.

Previous results have shown that despite DM females ovulating equivalent numbers of eggs to Controls at 6 weeks of age, ovaries contain a higher number of CL, albeit different in size and appearance (Grasa *et al.* 2012). In this study, we evaluated if the increased number of CL in 6-week DM ovaries is due to defects in CL regression. The CL present in the ovaries were counted and morphologically classified as Newly formed CL, coming from the last ovulation or Type I or Type II regressing CL (Control *n* = 9, DM *n* = 8) (Fig. 5A). The size of Newly formed CL in DM was similar to Control CL (Fig. 5B). However, the size of regressing Type I (*P* ≤ 0.0001) and Type II CL (*P* = 0.06) was smaller in DM ovaries compared with Controls. The number of CL was also evaluated and DM ovaries contained higher numbers of CL compared with Controls (10.1 ± 0.9 vs 17.3 ± 1.8, *P* ≤ 0.01). There was no difference in the number of Newly formed and Type I regressing CL between Control and DM. In contrast, DM ovaries contained 55% more Type II regressing CL (11.8 ± 2.5 DM vs 5.3 ± 1.3 Control; *P* = 0.06).

### Apoptosis of DM regressing CL is decreased

**Figure 6:**
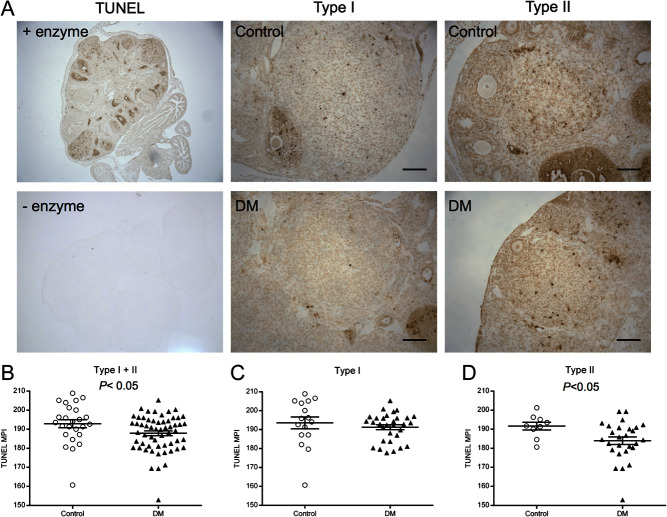
Assessment of apoptotic cells in CL. (A) Representative images of ovary sections subjected to the TUNEL assay with or without the enzyme, and of Type I and Type II CL subjected to the TUNEL assay from Control (*n* = 3) and DM (*n* = 4) ovaries. Scale bars correspond to 50 μm. (B) Mean pixel intensity (MPI) of TUNEL staining of Type I + II CL combined, (C) Type I CL (Control *n* = 16 and DM *n* = 31) and (D) Type II CL (Control *n* = 9 and DM *n* = 27). Individual points represent data for each CL and the error bar is mean ± s.e.m.

To determine whether the increased number of CL in DM ovaries is due to aberrant regression, apoptosis of luteal cells from regressing CL (Types I and II) was evaluated using the TUNEL assay (25 Control regressing CL, *n* = 3 mice and 58 DM regressing CL, *n* = 4 mice) (Fig. 6). There was a decrease in TUNEL staining of all regressing CL in DM ovaries compared with Control CL (Type I + II, *P* ≤ 0.05) (Fig. 6B). When Types I and II were analysed separately, TUNEL staining was found to be equivalent in Type I Control and DM CL (Fig. 6C) but decreased in Type II DM CL (Fig. 6D). These data reveal a defect in structural regression that would explain the higher number of CL present in DM ovaries.

## Discussion

Although several factors have been associated with POI, the mechanisms that cause ovarian dysfunction are poorly understood and therefore research is essential with the overarching aim of developing potential treatments. The DM mouse is a model of POI exhibiting a rapid deterioration of ovarian function from a subfertile state at 6 weeks to infertile at 9 weeks with POI by 3 months of age (Williams & Stanley 2011, Grasa *et al.* 2012). Therefore, the DM model provides us with the opportunity to investigate how the structure and function of follicles is modified during the onset of POI and the mechanism(s) involved in the reproductive dysfunction.

Follicle counts at both 6 and 9 weeks of age revealed increased numbers of primary follicles which, can be attributed to either overactivation of primordial follicles or a block in follicle development. As the increase in primary follicle number does not correspond with an increase in the number of developing follicles, this suggests that the disruption of glycans impacts either growth of follicles or their progression to advanced FSH-dependent stages.

Whilst ovaries from prepubertal DM mice at 3 weeks of age are grossly normal and contain follicles at all stages of development (Williams & Stanley 2011), we report here that follicle development in postpubertal ovaries is dysregulated. Recently, it has been identified that two waves of follicle activation exist within the mouse ovary, the first (prepubertal) and second (postpubertal) waves (Hirshfield 1992, Mork *et al.* 2012, Zheng *et al.* 2014). A recent study (Zheng *et al.* 2014) has shown that the first wave of follicles are responsible for the induction of puberty, the establishment of the hypothalamic–gonadal axis and are ovulated, thus contributing to early fertility. These are progressively replaced by follicles from the second wave, which last through the remaining reproductive lifespan. The decline in DM ovarian function is temporally similar to the demise of the first wave of follicle development. Indeed, at 3 months of age, when only the second wave of follicle development exists in the ovary, DM females undergo POI. Therefore, the phenotype of DM females could be due to defects in the postpubertal wave of follicle development; unfortunately, this hypothesis cannot be tested due to genetic incompatibilities between the DM model and the model used to track the two waves.

Ovaries from 9 week DM females contained fewer developing follicles, but of those present, a higher proportion were healthier as assessed by the TUNEL assay compared with Controls. This indicates that follicles that pass the developmental ‘block’ are more likely to survive, potentially due to decreased competition. Increased follicle survival may also be linked to the increased FSH levels (Williams & Stanley 2011), which promotes the survival of developing follicles (Dorrington *et al.* 1983). Oocyte-specific factors are also known to play a role in apoptosis and we have recently shown that ovaries generating oocytes devoid of core 1 *O*-glycans have a higher expression ratio of GDF9:BMP15 accompanied with reduced apoptosis (Williams & Stanley 2008, Grasa *et al.* 2015). Furthermore, mutations in *GDF-9* and *BMP-15* have been reported in some women with POI and are believed to play a role in ovary dysfunction (Dixit *et al.* 2006, Laissue *et al.* 2006) and hence we propose the high FSH levels and potential alterations in *GDF-9* and *BMP-15* could be promoting DM follicle survival.

Next, we assessed the structure of the surviving follicles to determine if these were morphologically normal. Secondary DM follicles had a thinner theca cell layer and an indistinct BL. At the secondary follicle stage, theca cells are recruited from the stroma and produce testosterone, which is crucial for the growth and development of the follicles (Young & McNeilly 2010). Therefore, a reduced theca cell layer at the secondary stage indicates a delay in their recruitment to the follicle. This in turn could affect follicle development and in combination with the decreased number of developing follicles found in DM ovaries could explain the low serum levels of testosterone found at 3 months of age (Williams & Stanley 2011).

Previously, we have shown that the lack of core 1 *O*-glycans on oocyte glycoproteins regulate the expression of ECM proteins and also the form and function of the BL (Christensen *et al.* 2015). Changes in the structure of the BL and the ECM proteins will likely alter the traffic of regulatory molecules and for the release of the oocyte at ovulation. The ECM protein laminin has been reported to play a role in luteinisation; however, the reports are conflicting. Rat GCs cultured with laminin were stimulated to produce progesterone and exhibited luteinising effects (Aten *et al.* 1995). However, when human GCs were cultured with laminin, progesterone synthesis was suppressed (Fujiwara *et al.* 1997). Thus, alterations in laminin levels and structure of the BL may prevent ovulation of the oocyte from preovulatory follicles, resulting in their premature luteinisation at 9 weeks of age.

Successful ovulation implies meiotic resumption and release of the oocyte, and the structural remodelling and luteinisation of the follicle. These processes are regulated by complex signalling between the somatic cells and the oocyte in response to an LH surge (Park *et al.* 2004, Shimasaki *et al.* 2004, Russell & Robker 2007). Alterations in signalling, as a consequence of modified structure/function of oocyte glycoproteins and their paracrine interactions, could result in premature luteinisation and ovulation would likely be compromised; this we observed in DM ovaries and has been reported in karotypically normal women with POI (Nelson *et al.* 1994). Previously, we have reported that 9 week DM females are infertile with scarce ovulations (Grasa *et al.* 2012). In this study, DM ovaries collected after ovulation contain LUFs, suggesting the absence of both *O*- and *N*-glycans results in defects in preovulatory follicles culminating in a failure to ovulate. The exact mechanism by which preovulatory follicles fail to ovulate is unclear. However, evidence that the high circulating FSH levels in DM females may be inducing luteinisation comes from an *in vivo* study by van de Lagemaat *et al*. (2011), who demonstrated oral administration of short-acting FSHR agonist induced luteinisation of preovulatory follicles in rats, guinea pigs and cynomolgus monkeys. It has been suggested that the FSHR agonist mimics the rise of cAMP in GCs observed following the LH surge (Richards *et al.* 1998) and thus induces a suboptimal ovulation that results in the generation of a LUF. Indeed, it has been established that the actions of FSH are mediated through secondary messengers including cAMP (Robker & Richards 1998, Sirard *et al.* 2007) and therefore we speculate that the high circulating FSH levels in DM are responsible for the ovulation failure, resulting in the production of LUFs.

In this study, delayed CL regression in DM ovaries was found to be due to attenuated rates of luteal cell apoptosis, which would explain the high number of CL observed in DM ovaries (Grasa *et al.* 2012) since ovulation rate is equivalent at this age (Grasa *et al.* 2012). The fact that luteinised abnormal structures are found in 3 month DM ovaries accompanied by normal progesterone serum levels, despite the lack of ovulations (Williams & Stanley 2011), would indicate that functional regression is disrupted in these mice and luteal cells from the CL remain steroidogenically active. Regression of the CL or luteolysis begins with the loss of ability to produce progesterone followed by the loss of the cells that form the CL (Niswender *et al.* 2000). CL regression is required for normal reproductive function, which is not the case in the DM (Williams & Stanley 2011), and therefore it is possible that the abnormally regressing CL have a role in DM reproductive dysfunction. Furthermore, FSH plays a role in GC proliferation, survival and differentiation (Richards *et al.* 1995) through the PI3K pathway (Alam *et al.* 2004). Targeted disruption of an inhibitor of the PI3K pathway, Pten, in GC resulted in the persistence of CL as a result of the increased lifespan of luteal cells (Fan *et al.* 2008). Therefore, it is possible that the high FSH levels in DM females mediate activation of the PI3K pathway resulting in the persistence of CL by extending the lifespan of luteal cells.

In this study, we also determined that despite the infertility, DM females are sexually receptive. This also suggests there is some ovarian steroidogenic activity present in DM females and also challenges the current paradigm that follicle development tightly regulates reproductive cyclicity. It is well known that oestrogen regulates sexual behaviour by acting on the brain (Ogawa *et al.* 1998, Flanagan-Cato 2000, 2011) and is responsible for the cornification of the vaginal epithelium observed during the oestrous phase. One possible explanation for the reproductive behaviour of DM females despite abnormal follicle development, could be that as the CL produce oestrogen, estradiol serum levels are not modified in these females suggesting that at least some signalling between the hypothalamic–pituitary–gonadal axis remains intact and so regular cycles remain.

In conclusion, we have investigated the aetiology of POI in a mouse model of follicular POI and have revealed multiple defects in follicle development and ovarian function before the presence of the POI phenotype. Although rodent ovarian function and luteal control is different to women, many of our reported findings have already been observed in women suffering from POI. Thus, identifying these follicular modifications have provided insight into the mechanisms driving the development of POI and highlight the opportunity the DM mouse model offers to investigate the pathogenesis of POI and potentially to develop new fertility treatments.

## Declaration of interest

The authors declare that there is no conflict of interest that could be perceived as prejudicing the impartiality of the research reported.

## Funding

This work was supported by a grant awarded from the Medical Research Council to S A W (G090058). S S is a recipient of a Leverhulme Postgraduate bursary and EPA Cephalosporin Scholarship from Linacre College, University of Oxford. N K, K M, S J, and P N were partially funded by the NDOG.
